# Welfare Through Competence: A Framework for Animal-Centric Technology Design

**DOI:** 10.3389/fvets.2022.885973

**Published:** 2022-06-30

**Authors:** Sarah Webber, Mia L. Cobb, Jon Coe

**Affiliations:** ^1^Faculty of Engineering and Information Technology, School of Computing and Information Systems, The University of Melbourne, Parkville, VIC, Australia; ^2^Faculty of Veterinary and Agricultural Sciences, Animal Welfare Science Centre, The University of Melbourne, Parkville, VIC, Australia; ^3^Jon Coe Design, Healesville, VIC, Australia

**Keywords:** animals, animal-computer interaction, animal-centric design, animal welfare, animal technology, interaction design, digital enrichment

## Abstract

Digital technologies offer new ways to ensure that animals can lead a good life in managed settings. As interactive enrichment and smart environments appear in zoos, farms, shelters, kennels and vet facilities, it is essential that the design of such technologies be guided by clear, scientifically-grounded understandings of what animals need and want, to be successful in improving their wellbeing. The field of Animal-Computer Interaction proposes that this can be achieved by centering animals as stakeholders in technology design, but there remains a need for robust methods to support interdisciplinary teams in placing animals' interests at the heart of design projects. Responding to this gap, we present the Welfare through Competence framework, which is grounded in contemporary animal welfare science, established technology design practices and applied expertise in animal-centered design. The framework brings together the “Five Domains of Animal Welfare” model and the “Coe Individual Competence” model, and provides a structured approach to defining animal-centric objectives and refining them through the course of a design project. In this paper, we demonstrate how design teams can use this framework to promote positive animal welfare in a range of managed settings. These much-needed methodological advances contribute a new theoretical foundation to debates around the possibility of animal-centered design, and offer a practical agenda for creating technologies that support a good life for animals.

## Introduction

Digital technologies offer new ways to ensure that animals in human care can lead a good life across a wide range of contexts. These contexts include, for example, zoos, farms, domestic settings, kennels, stable facilities, veterinary hospitals, animal shelters, research facilities, and wildlife sanctuaries. Such sectors are rapidly increasing the use of animal-centric devices such as wearable tracking devices, digital enrichment, automated feeders, robotic gates, and milking machines. In many settings is also common for carers and other humans to use technological devices as part of animal management and care, which may impact on animals and on human-animal interactions. These include data gathering devices, veterinary equipment, communication systems including screens and audio-visual equipment. However, in designing for animals' physical and mental wellbeing, a significant challenge lies in “centering” the animal—that is, identifying and prioritizing design objectives from the animal's viewpoint. Historically, design for animals has been guided by the drive for efficiency, economic gain, the preferences and goals of human carers, and by tradition or common practice, rather than by understandings of animals' ancient evolutionary nature and welfare needs. For example, although zoos have a long history of creating naturalistic environments, their design is often heavily influenced by visitor experience objectives, the practicalities of cleaning and animal management, and by the practice of imitating or improving on existing exhibits at other zoos. In the animal production sector, technological innovation may respond primarily to industry standards and to commercial pressure to increase efficiency and productivity.

Animal-centric goals are essential as a focus in design projects, to ensure that technology interventions' outcomes promote life-long mental and physical wellbeing for animals. The emerging discipline of Animal-Computer Interaction (ACI) proposes that design of technologies can achieve an animal-centered orientation and uncover new opportunities for technology to contribute to a good life for animals, by adapting the processes and methods of interaction design, as used in human-centered innovation projects (1–3). However, a core challenge of this approach lies in identifying what animals “need” or “want” (2). On the other hand, animal welfare scientists and designers of environments for animals (such as zoos, shelters, farms, and kennels) have a strong understanding of what is required to address animals' essential welfare needs but may miss opportunities to deploy design practice as a way to learn more about animals' preferences and about the potential benefits of digital technologies to animals. Increased understanding of animal sentience has highlighted the importance of enabling animals to be active agents in their lives. This emphasizes the need for opportunities for animals to develop independent competence within managed settings, which technology can support in novel ways. Our aim is to build on and advance ACI research toward a methodology that includes animals as key collaborators in multi-stakeholder design projects. To date, recommendations for conducting animal-centric design projects have been fragmented, and have not addressed the critical issue of how animals' wellbeing goals can guide a project from its outset. The purpose of this paper is to address this gap by proposing a framework for designing technology to promote a good life for animals, which integrates interaction design practice with models of animal welfare and design for animal competence.

### A Good Life for Animals

To successfully design for animal wellbeing, a clear understanding of what constitutes a good life for animals is required. The concept of animal welfare can be considered equivalent to quality of life and wellbeing; an animal's welfare status is informed by many facets of its life and can vary from very poor to very good (4). The subjective experience of an individual animal is influenced by how the conditions in which it lives impact its affective state (5–7). That is, what does the animal need to do to cope and thrive in life, and how does that make the animal feel? Considering positive welfare, or a good life for animals as “enjoying good health (having what they need) and having access to what the animals themselves want, while also liking what they have” provides a modern, animal-centered perspective on what is meant by *animal welfare* (8).

Recent advancements in animal welfare science demonstrate evidence from areas such as neuroanatomy, comparative cognition, and physiology that has established the sentience of animals. This means that vertebrate animals and a growing number of invertebrate animals (e.g., octopus and lobster) can consciously experience awareness and different feelings such as pain, joy, frustration, loneliness and comfort. Understanding that animals are sentient requires us to identify the needs of animals as a significant moral obligation. This is particularly true for animals kept under human care, where environmental, social and behavioral opportunities are often restricted (9, 10). This is reflected in the recent recognition of animal sentience in legislation globally, and shifting community attitudes of concern toward animals and the industries that manage or interact with them (11). These new understandings of animals' sentience imply that humans should ensure that the animals they care for enjoy a good life, going beyond the minimization of negative experiences such as harm or discomfort. As part of this shift toward ensuring positive experiences, there is growing attention to the value of exercising agency, building competencies and appropriate levels of challenge as important contributors to wellbeing of animals in human care (12–14).

Historically, the management of animals has been anthropocentric. Across modern animal care settings, the attitudes and consequent behaviors of people responsible may not align with the animal welfare evidence base or animal preferences (15). In response, some people have proposed that the animals must change to cope with the settings people have placed them in; that there is a need for animals to be *resilient* to cope with welfare challenges and *robust* to maintain productivity without compromise (16). An alternative strategy, which aligns with the change in community attitudes toward animals, is to seek new ways to care for animals in managed settings that prioritize their wellbeing. Where practices relating to animal care and management have been shown to conflict with community expectations, industries have experienced significant interruption or termination of their social license to operate (17). For example, community concerns about animal welfare played a substantial role in the reshaping of zoos (18) and have recently had considerable impact on the use of exotic animals in circuses (19) and greyhound racing (20). Similar shifts are now occurring in public attitudes regarding farm animal welfare (21), which has substantial implications for the sustainability of animal production (22, 23). The importance of promoting positive welfare has relevance for the horse racing sector (24) and for working dogs, as reflected in official standards for security and detection dogs (25). Taken together, these points highlight the need to provide all animals under human care with a good life, by creating environments, equipment and systems centered on animals, aligning with community expectations and modern scientific understanding of critical factors such as animal sentience.

### Designing for Animal Wellbeing

Sectors in which animals are managed, notably zoos and farms, have a history of designing built environments to meet essential welfare needs. Standards of care for animals' environments have traditionally been based on the “Five Freedoms” principles of animal welfare, which arose from the livestock-focused Brambell Report (26, 27). The Five Freedoms principles aimed to provide animals with “freedoms *from”* negative conditions and suffering: 1) Freedom from hunger and thirst and malnutrition (through ready access to fresh water and adequate diet to maintain full health); 2) Freedom from discomfort (by providing an appropriate environment that allows for shelter and rest); 3) Freedom from pain, injury or disease (by preventative health care and/or provision of rapid diagnosis and treatment); 4) Freedom to express normal behavior (through provision of sufficient space, resources and social interaction); and 5) Freedom from fear and distress (by providing conditions and treatment which avoid mental suffering). These principles guided the provision of a minimum baseline of acceptable welfare which should be met in the design and management of farms and other settings.

As scientific understanding and societal concern regarding animal welfare have grown in recent decades, new methods to consider and assess animal welfare have emerged. These modern methods have influenced the provision of environments and resources in some settings, including good, modern zoos (10, 28). The current animal welfare models recognize that animals should feel well, be biologically functional and lead reasonably natural lives (29, 30). The assessment of animal welfare is today most commonly informed by the structured Five Domains of Animal Welfare Model (7). The Five Domains offers a systematic way to assess indicators of internal and external physical and functional states, environmental conditions and how these then influence the subjective mental experiences of animals. Unlike the older Five Freedoms, the Five Domains Model combines “freedom *from*” and “freedom *to*” by considering both negative and positive mental states. This model provides a valuable tool for assessing and remedying existing facilities and programs, but does not specifically address the issues of promoting animal competence, generating animal-centric design requirements or including animals in design practice.

There has been increasing recognition that species-specific strategies and interventions are needed to improve the lives of animals. Such strategies include being required to “work” for their food (31), creating opportunities to exercise highly-motivated natural behaviors, variation of the environment and addition of sensory stimuli, and allowing animals some degree of independent control over their lives. Many of these strategies can be achieved through “environmental enrichment,” which takes many animate and inanimate forms, including “occupational” enrichment such as food puzzles, control of the environment and physical exercise; “physical” enrichment from interacting with complex environments including structures and appropriate substrates; “sensory” enrichment, including visual, auditory, and olfactory stimuli; “nutritional” enrichment through variation of delivery, food type and challenging presentation; and “social” enrichment comprising interactions with other animals and with humans (32, 33). In recent decades, zoos, aquariums, and similar facilities have pioneered the design, creation, and evaluation of enrichment (34, 35). The use of enrichment has demonstrated benefits to animal welfare for animals living in a range of contexts, including laboratory, farm, zoo, aquatic, and kennel environments [e.g., (15, 32, 36–38)]. Important considerations in the design and provision of enrichment are that it should be relevant to the animal's “behavioral needs” (39), and context (40), and that it should provide appropriate levels of challenge (41). Enriched and challenging environments play an important role in enabling animals to gain competence, including flexible problem-solving skills and mastery in specific tasks (12). To develop competence and exercise agency, an animal must be exposed to novelty, broad sensory experiences and opportunities for learning through interaction. Through gathering environmental information, exposing themselves to risk and training their capabilities through exploration and play, animals build the ability to solve problems that are meaningful to them, with respect to their ecological niche (12). Indeed, opportunities to develop competence may play a significant role in addressing ethical and welfare concerns associated with keeping animals in managed settings (14).

The potential for technological devices to contribute to animal wellbeing was explored as early as the 1970s, notably by Markowitz and colleagues at Portland Zoo. As cognitive enrichment for primates housed in barren environments, Markowitz created installations which required animals to push specific buttons or levers in response to artificial stimuli such as lights, or work to obtain tokens which could then be exchanged for food rewards (42). Operant training was used to shape animal behavior and teach animals how to play the games (42). In cognitive research programs, primates and other animals have long used technologies such as joystick-controlled computers and touchscreen interfaces (43, 44), and there are claims that this type of activity can be enriching for animals (44). In recent years, researchers have explored the potential of using sensor-based technologies to provide animals with greater variety, and more opportunities for active interaction and agency in their environment (45–48) and to offer substitutes for natural behaviors, such as hunting live prey (49). In parallel, there has been a rapid uplift in the potential for conducting digital monitoring and tracking of animals in zoos, farms and other settings, using animal-attached sensors, bioacoustics (50), video-based analysis (51, 52), and other technologies embedded into animals' environments. Technologies such as precision agriculture systems are generally grounded in the needs of human stakeholders and the aims of improving efficiency and productivity, but can also contribute to animal welfare goals (53), and be designed with consideration of the needs of animal stakeholders (54).

### The Challenges of Animal-Centric Design

With the increasing use of digital technologies for and with animals in a range of settings, there is an urgent need for technology design methods which can account for and respond to the needs and interests of animals and, at a minimum, ensure that there are no negative impacts on animal welfare. The field of ACI addresses this challenge, investigating how animal-centric digital technologies can best contribute to a good life for animals, and to develop relevant methods and theories to achieve this. A commonly cited aim, formulated by Steve North, is that ACI would “build only what they [animals] want or need” (55). Researchers from animal sciences have drawn attention to the breadth of opportunities that interactive technologies offer for enhancing animal welfare (56). However, there is a risk that such interventions can introduce unintended harms, or inadvertently promote misconceptions about animals' needs, if they are created without inclusion of appropriate expertise and careful attention to the genuine needs and interests of the animal (59). Limited attempts to include the perspective of the “non-verbal other” (60) in design entails the risk of attributing desires to an animal which correspond with preconceived notions of what animals need or want (59). North has also drawn attention to the risk of “unconscious projection of personal design priorities and enthusiasms” onto “voiceless co-designers” (55) as part of a call for robust, interdisciplinary methods for ACI design and research.

As an interdisciplinary field, ACI has applied a variety of theoretical and methodological lenses to the question of how we can elicit and respond to animals' requirements in designing digital technologies. Mancini's manifesto proposed that user-centered design methods, commonly used by interaction designers and human-computer interaction researchers, could be used alongside methods and knowledge developed in animal sciences, to access an animal's perspective (1). One approach for formalizing an animal-centric design process, the “Agility, Welfare as value and Animal eXpert involvement” model (AWAX), was devised by van der Linden and Zamansky (61). The AWAX process is initiated with specifications created by a technical team, rather than starting with what is known about the animal to identify design opportunities and objectives. French et al. draw on their experience in designing for elephants and other animals to propose a deck of “Concept Craft Cards” which are intended to support designers in envisioning ACI interventions, with prompts related to aesthetics, species characteristics, values and user experience (62). To leverage scientific and zoo-based knowledge in defining design objectives, Veasey proposes the “Animal Welfare Priority Identification System” (APWIS), an approach based on the Delphi method, in which animals' needs are identified and weighed by a panel of species experts and specialists (63, 64). It remains unclear however, how animals' needs might be incorporated into an iterative design project, or revisited as new knowledge emerges through the design process.

ACI designers face substantial challenges in imagining how an animal will respond to new interactive opportunities (65), and in crafting experiences which will be aesthetically interesting to animals (62) and be of ongoing interest and benefit. Many ACI researchers seeking to enhance animals' lives have found that animals respond to novel interventions with disinterest (66), fear (67), or active destruction (68). Such investigations can be costly and time consuming if they entail extensive work to ensure that hardware components are safe and sufficiently robust for animal use (48, 68) or require considerable training for animals to use them successfully (67). Even ACI installations which are initially used successfully can fall into disuse, suggesting that they require modification to provide ongoing meaningful benefits to animals (46, 69). This suggests that there is a need for methods which will guide designers in identifying appropriate solutions while minimizing effort and cost spent on exploring alternatives which might not be beneficial or successful.

There remains a need for structured approaches that guide interaction designers, animal scientists and carers to systematically explore animal wellbeing design opportunities, convert them into animal-centric design objectives, keep them in focus and refine them through the course of a design project. It is notable that the interdisciplinary nature of animal-centric design means that methods and tools should be accessible to interaction designers, animal experts and carers, and should help teams to communicate and collaborate despite differences in methodological backgrounds. In this paper, we draw on our cross-disciplinary experience of designing and evaluating interventions for animal welfare, and extend the prior work of designers (62), animal welfare experts (63), and computer scientists (61) to present the Welfare through Competence framework (WtC), grounded in interaction design practice, animal welfare science and expertise in world-leading zoo design. This framework is offered as a guide and support for teams of practitioners and researchers aiming to create technologies that contribute to a good life for animals.

## Materials and Equipment

In this section, we introduce the existing models of animal welfare and competence, design for animals and interaction design that we employ as a foundation for animal-centered design. As a contemporary model of animal welfare focused on identifying positive welfare outcomes, we adopt the Five Domains model developed by Mellor, Beausoleil and colleagues, most recently presented in Mellor et al. (7). We complement this with the Coe Individual Competence Model (70, 71), which provides designers a structure for thinking about the long-term needs of animals in managed settings and which emerged from extensive design work in zoos and sanctuaries. The third model we deploy is the interaction design cycle, an approach to iterative, user-centered prototype-based design widely used by design practitioners and human-computer interaction researchers.

### The Five Domains of Animal Welfare Model

The Five Domains of Animal Welfare Model (7) is widely recognized as a paradigm for systematic consideration of how animals' wellbeing relates to their lived experiences. The Model assesses indicators of welfare across the physical and functional domains of (1) **Nutrition**, (2) **Environment**, (3) **Physical Health**, and (4) **Behavioral interactions**, which together inform the final domain, (5) **Mental State**. In addition to placing Mental State at the center of animal welfare considerations, the Five Domains provides a Model that observes positive states with equal emphasis as negative states. In assessing an animal's welfare, the Five Domains Model relies on behavioral and physical indicators and resource provision to infer animal wellbeing. This acknowledges that animal welfare is experienced subjectively at the level of the individual; we must rely on these indicators as the absence of validation and consensus mean that we are not yet able to directly measure an animal's subjective welfare (72, 73). The success of the Five Domains Model is evidenced by its widespread international adoption, including by organizations such as RSPCA UK and the Zoo and Aquarium Association Australasia. It is a valuable tool for the systematic examination of the different aspects of an animal's present experiences. However, it does not provide a process for envisaging possible futures or generating solutions to deficiencies, other than identifying improvement in mental state as an indicator of success. While not a fault of the Five Domains Model, suggested solutions to compromised welfare tend to be framed as: “What can people do for the animals to improve their welfare in this domain?” rather than empowering animals to competently improve their own welfare as they prefer.

### The Coe Individual Competence Model

The Coe Individual Competence model (71) was developed to provide a practical agenda for designers of managed animal facilities and enrichment to create environments which enable animals to develop competence as part of positive animal welfare. Competence can include broad capacity to address challenges and novel problems, as well as mastery in specific tasks (12). This model responds to the growing body of research which attends to competency and agency in animal wellbeing (13), and the significance of factors such as novelty, predictability, complexity, challenge and sensory experience (12).

The Coe Individual Competence model calls for animals to be offered opportunities which entail (1) **Choice**, (2) **Control**, (3) **Variety**, and (4) **Complexity**, which all contribute to the development of (5) **Competence** (71). With a focus on *freedom to* rather than *freedom from* as also described in the Five Freedoms principles, the Coe Individual Competence Model provides a structure to identify enrichment opportunities that can contribute to animals' ongoing development of physical, social, and mental capabilities for living a good life. Ideally, levels of competence can be compared to, but not limited to, species-typical natural behaviors recorded in the wild. For example, zoo-housed orangutans should have the strength and dexterity to be agile climbers as wild conspecifics are, but may also become competent to use symbolic language on a touchscreen computer interface if they wish. This approach is based upon providing animals with an enabling environment, and supportive training and conditioning opportunities, to develop necessary levels of competence and agency to benefit from the increased opportunities listed here. We have defined each of the foci of this model for clarity as follows.

#### Choice

Choice entails having opportunities to select between two or more options. Choice is provided by enabling animals to make decisions and encouraging animals to exercise agency (13). As well as being inherently rewarding (14), opportunities to choose can enable animals to address their physical, homeostatic needs, as in the case of having nutritional choices (74), and to develop the competencies they may need to access desired resources in future (13). Managed settings often restrict animals' freedom of movement and other behavioral choices (e.g., social interaction and breeding opportunities), but can be designed to provide greater access to preferred environments and experiences as compared to wild settings (14). Choice is often based upon relative, rather than absolute preference. Choices offered to the animal should remain within the limits of what may be helpful and not harmful.

#### Control

Increased control or agency has been proven to improve welfare (13, 75). It entails giving animals the power to influence (limit, order, or direct) behavior, actions, environment, or the course of events, enhancing individual capacity to cope with novel problems. Control is provided when animals can actively decide when, how, where and/or with whom to interact without external interference. While many animals under human care lack opportunities for control, they can derive inherent benefit from features specifically designed to allow them to say, activate showers, trigger food delivery, or change lighting (42, 70, 76). Exercising control and agency is intimately linked to developing competency, in that it enables animals to gather knowledge, develop novel behaviors and enhance skills through exploration and play, instrumental or social learning, and communication (13). The term “competency-building agency” has been used to denote a level of agency which may not deliver immediate outcomes but enhances animals' capacity for, say, more efficient foraging, or addressing future challenges (13). It should be noted that an animal choosing *not* to use an enrichment feature or other intervention is also exercising agency. Coe (77) has suggested that the organisms with the greatest degree of choice and control have the greatest degree of relative freedom.

#### Variety

Variety involves experiencing quality or states that are diverse. In the wild, animals move through a varied spectrum of environments to meet their needs and are likely to encounter changes with the seasons and over time. Variety entails novelty and is a prerequisite for building competency (12). Facing new objects, situations, events and challenges encourages “inspective exploration” and “inquisitive exploration,” and enables animals to develop flexible problem-solving abilities (12). For animals in human care, variety can be introduced by incorporating a range of physical and sensorial features, making alterations over time, or by providing access to multiple, different spaces. Variety can also take the form of different foods, varied social opportunities, and opportunities to exercise a variety of behaviors—for example, using different foraging strategies.

#### Complexity

Complexity involves engaging with many interrelated parts (e.g., objects, ideas, activities, environments, etc.) that may be connected in intricate and complicated ways, with no simple solution. Complexity is provided when animals find situations or tasks challenging to analyse, understand or solve and rewarding to achieve (41). The challenges presented by rich, complex and unpredictable environments demand ongoing learning. From an animal's perspective, a complex environment that is also variable is highly probabilistic: repeated attempts and intense engagement may be required to access resources, and this provides a setting where animals can build mastery and perhaps come to detect hidden contingencies (12). Animals evolved to prosper in an often-changing complexity of physical and social environments, and welfare can be compromised through boredom leading to frustration and a lack of physical conditioning and mental acuity (32).

#### Competence

Competence is gained by animals who achieve the functional abilities (physical and mental, innate, and learned) to be able to realize desired outcomes effectively and efficiently. In an evolutionary sense, the successful wild animal is *de facto* physically, mentally, and socially **competent**, exercising **choice** and **control** amid a wide **variety** of **complex** physical and social environments to achieve success. We suggest that offering animals in human care opportunities to engage and gain skills in these areas will help them to better manage their own lives through increased agency and competence (73). Competence requires the development of capacities along several dimensions: physical, social, cognitive capacity, knowledge acquisition (12). It allows for problem-solving and achieving desired outcomes in the short and long term. Developing competence is life-long and incremental, and need not be rushed. Competence along any of the above focal dimensions (exercising Choice or Control, and responding to Variety or Complexity) may take time to acquire, depending upon the physical, social and management environments. Progress toward competence can be evaluated using the Five Domains Model. Competence to achieve a high level of physical, mental, social, innate and learned abilities may be considered the measure of the self-actualized animal (71) in nature or in managed care (78). Competence is the set of demonstrable characteristics and skills that enable increasing agency and self-determination for the animal resulting in a good life.

**Time** and timing (occurrence, frequency and duration) is a factor or opportunity for consideration in each of the competence focal categories (79). For example, **Choice** of timing, frequency and duration of enrichment occurrence; **Control** of timing of occurrence, frequency and duration; **Variety** and **Complexity** of timing options could all support development the animal's **Competence**. Ideally, enrichment opportunities should be scheduled to coincide with the species' natural circadian rhythms rather than caregiver working hours. However, success of interventions also requires that they be feasible for animal caretakers to implement—a factor which is associated with workplace satisfaction and caretakers' mental health (80).

The design of interventions for animal competence should account for the prior experiences and competence of the animal, and should consider how competence can be augmented over time. As part of this, it is important for designers to consider how new animals will be introduced to environments in which ACI interventions have been deployed; for example, habituation, training and incremental introduction may be required. In addition, it is important to consider the exit process for animals who will be removed from an enriched environment to one in which they have lower levels of choice, control, variety or complexity.

### The Interaction Design Process

Building on the approaches suggested by ACI scholars (1, 55), we adapt and extend interaction design methods, which are widely used to create user-centered digital technologies such as interactive websites and mobile applications (81). The process of interaction design entails learning about the future users of a digital product, using these insights to define what should be built, creating prototypes (rough representations or approximations), and evaluating these prototypes. A key aim of interaction design methods is to gain input and feedback from potential users and other stakeholders throughout the process. The steps are often portrayed as a design “cycle” which should be conducted iteratively, on the basis that information gained through testing early prototypes can give designers important new understandings of what should (or should not) be built, and how to build it successfully (82). This iterative approach to design contrasts with “waterfall” development methods in which all research and requirements gathering is performed upfront, before design takes place (83).

Interaction design commences with understanding users, or developing “empathy,” and learning about the problem space, i.e., the nature of the goals and issues faced by potential users, the limitations of existing solutions, and the context of use. This information, collectively, is used to *define objectives* for the product, i.e., to determine what should be built. Designers are encouraged to undertake creative *ideation*, generating multiple alternative ideas about how the objectives could be addressed through e.g., brainstorming. Ideation, especially if conducted in collaboration with future users and stakeholders, can lead to new insights about the problem, enabling the team to refine the objectives. From these candidate ideas, one or more will be selected as the basis for *prototyping*. As part of the iterative process of learning, the first prototypes are created with the intention that they will be thrown away, and so should be low-cost and quick to create. Early prototypes may include storyboards (84), paper prototypes (85) and “Wizard of Oz” solutions, in which the role of a future system is played by a human operator (86). These are often referred to as “low fidelity” prototypes (87): they look very different from the envisaged product they represent, but can still allow people to give valuable feedback about what a product will deliver, and how it will be used. Later on (after a few iterations of the interaction design cycle), design teams are likely to turn to software and interactive devices to create “high fidelity” prototypes, which look and behave more closely like the envisaged product (85).

Evaluation of prototypes can result in many different forms of insights, which may be used to redefine the objectives, to generate new design ideas, or to inform subsequent prototypes. Evaluation conducted in early iterations of a human-centered design project often takes the form of workshops, focus groups and walkthroughs, aiming to validate the overall aims of the project and learn more about alternative design directions. Through user evaluation, teams may well learn more about the users and the problem space. Subsequently, “formative” evaluation of higher fidelity prototypes is likely to focus on improving usability and the “user experience” (82, 88), but it generally becomes more costly to make significant changes to a product proposal (83). Late-stage evaluation of candidate products may also investigate the effectiveness of alternative designs in terms of, say, productivity (in the case of a workplace application), or learning outcomes (for an educational tool). “Summative” evaluation seeks to establish the effectiveness of a final candidate product and is often conducted with users immediately prior to a product being deployed (89, 90).

**Figure 1:**
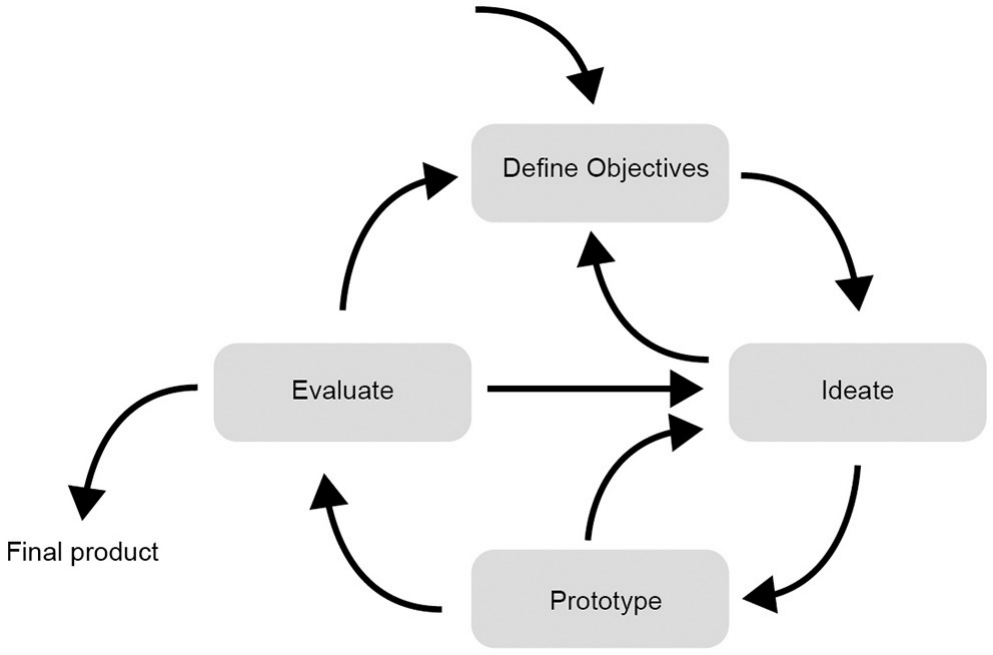
An iterative interaction design process.

There exists great variety in the way that interaction design processes have been defined by institutions such as Stanford's d.School, Google and IDEO (91–93). Processes vary in terms of the number of steps (commonly, 4–6 steps), the terminology used, and graphical representations. We have adopted a simple design process, employing terminology often encountered in interaction design and allied fields of user experience and human-computer interaction. A graphical representation of this process is shown at Figure 1. This diagram represents a broadly cyclical process entailing four activities: *define objectives, ideate, prototype, evaluate*; and also shows that information and ideas can flow in different directions between the four activities.

Many ACI scholars have recognized the potential benefits of iterative, user-focused interaction design methods as a means to include the “voice” of animals in creating a product, and “center” animals' needs, and thereby avoid misguided, and anthropocentric design directions (3, 65, 94, 95). However, our own experiences and those of other ACI designers indicate that there are several challenges to including animals in interactive, prototype based design processes (96). Firstly, animals cannot verbally express their goals and desires, and so designers often struggle to identify and prioritize animals' essential needs to inform design objectives (57, 59). Secondly, prototypes can be rapidly destroyed by animals (97), so may need to be replaced repeatedly or constructed in highly robust fashion, which can be costly and time-consuming. Thirdly, it is problematic to make inferences about how useful or suitable an intervention is to animals based on their initial responses (98) as their early interactions are likely to be shaped by the impetus toward inquisitive exploration (13) and the “novelty effect” (99), or by neophobia or startle responses (67). Fourthly, methods of evaluation used in animal care sectors can conflict with iterative design approaches in which prototypes are repeatedly changed in response to formative evaluation (96). Our aim is to adapt interaction design methods to overcome these challenges, providing a lightweight, learning-focused process (as set out in Section Applying Interaction Design Process) for ACI design teams to maximize attention to animals' needs in the context of the WtC framework.

## Methods

**Figure 2:**
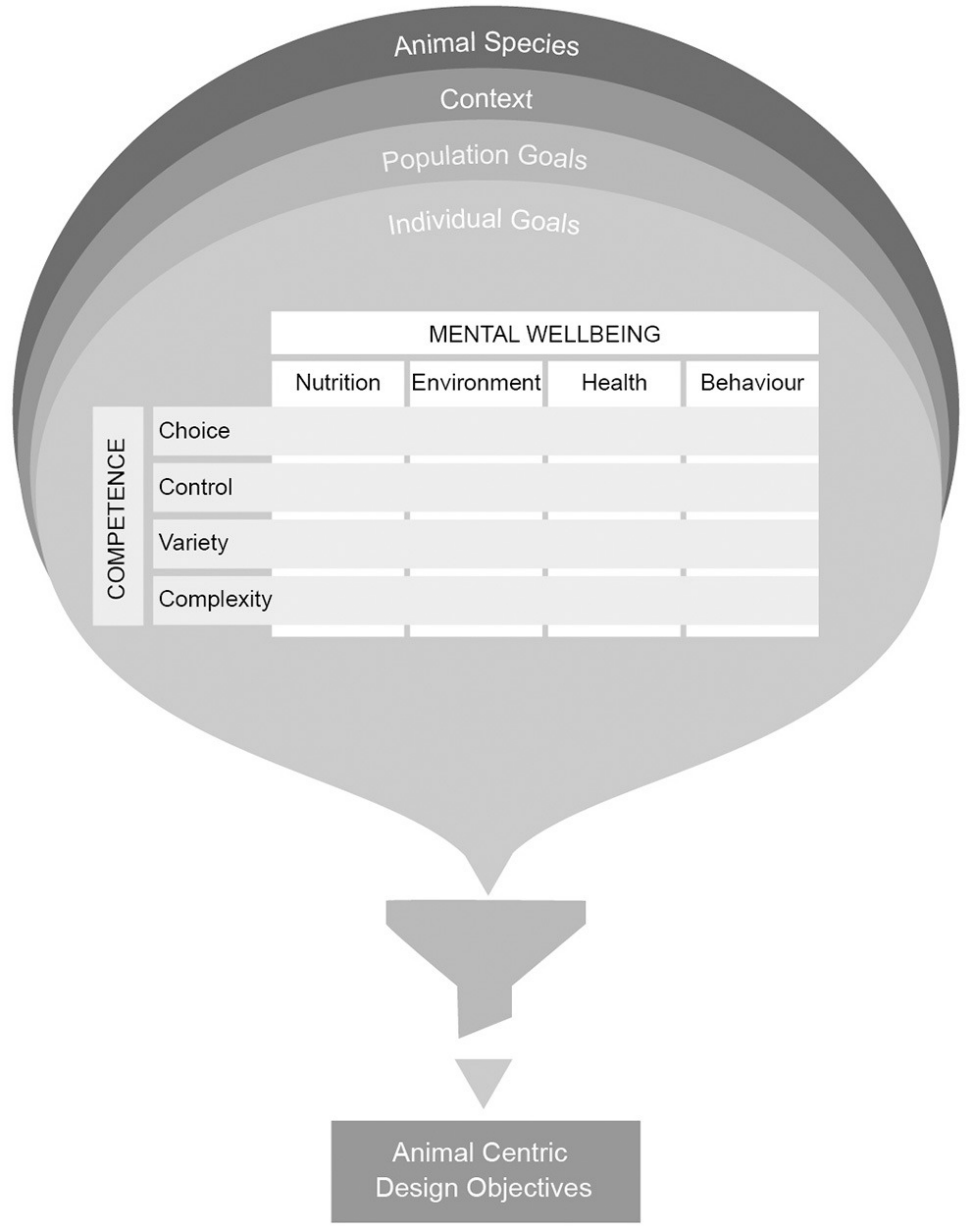
The welfare through competence animal objectives canvas.

**Figure 3:**
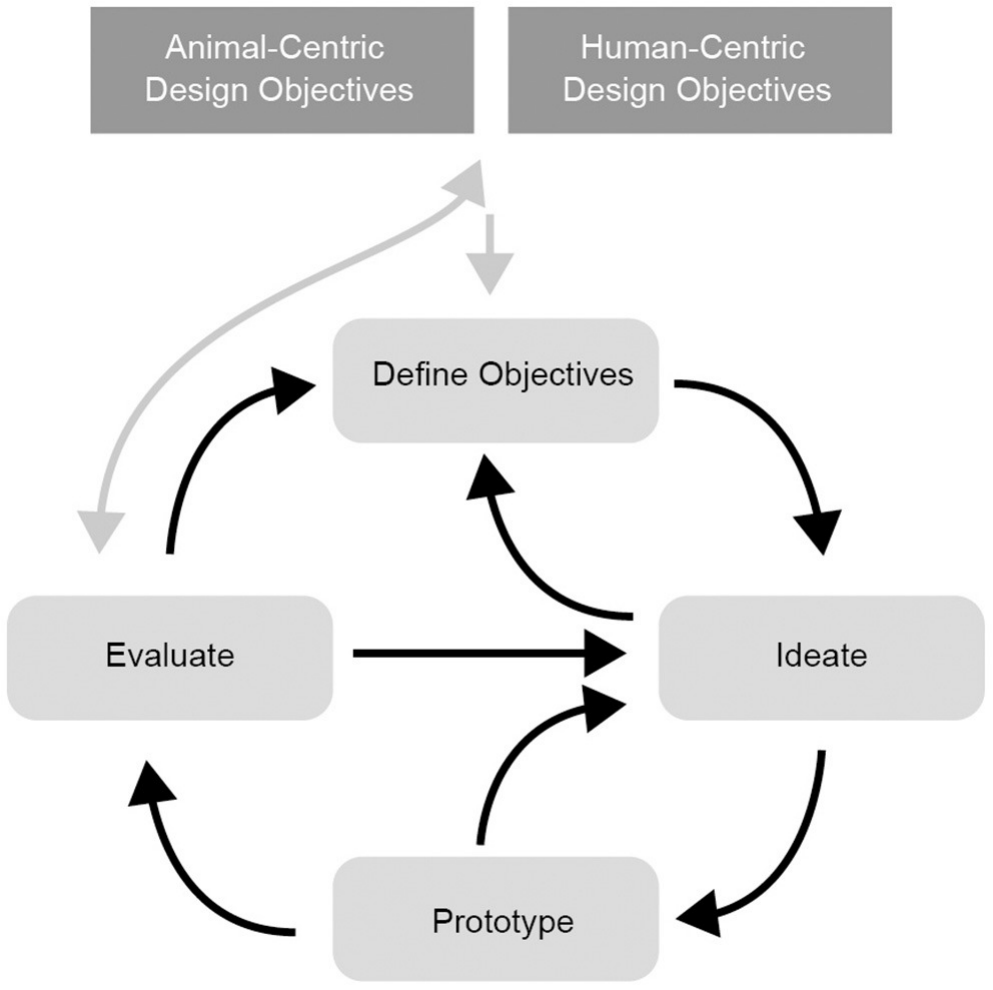
The welfare through competence interaction design process.

Responding to the challenge faced by ACI designers in identifying what it is that animals want and need, we present the WtC framework for design centered around a matrix which synthesizes the Five Domains model and the Coe Individual Competence model. The framework prompts designers to identify animal-centric design opportunities using the matrix, giving consideration to the individual animal, its group population, the context and the animal species. The WtC Animal Objectives Canvas, represented in Figure 2, provides a structured approach to define animal-centric design objectives as input to a technology design project. We describe how these objectives can be included in an Interaction Design process shown at Figure 3, in such a way as to validate, refine or redefine the animal-centric objectives and revisit them through the course of a design project.

### The Welfare Through Competence Design Opportunities Matrix

Synthesizing the Five Domains and Coe Individual Competence models into a matrix, as shown in Table 1, provides a basis to assess and identify opportunities for a good life for animals. We propose that designers can systematically explore animal-centric design opportunities by considering how each of the competence foci (per the Coe Individual Competence model) can contribute to each of the Five Domains of animal welfare. For example, designers might first consider how each of the Competence foci can play a role in supporting an animal's welfare in the domain of nutrition, as follows:

- Considering the focus area of **choice** and asking what food and feeding options would the animal have in the wild might reveal opportunities for providing greater, more natural choice of food such as seasonally available variations (while still ensuring that the animal has a nutritionally complete and balanced diet) or feeding schedule (single predictable feeding or multiple random feedings), which would the animal choose? Which would be best suited to its evolutionary adaptations?
- Designers would then progress to consider goals related to animal **control** in the domain of nutrition. How would the animal choose to control food recourses and availability choices within a healthy diet? For example, would the animal prefer to control a food delivery mechanism itself rather than having the same food delivered by a caregiver?
- Opportunities for **variety** in nutrition might be considered next, including mechanisms for varying the schedule, location, type and quantity of food available from day to day, or according to the season (while ensuring that nutritional needs are met). Designers might consider aspects of timing, including the frequency with which a given food is presented, and varying the interval between feeding events.
- In considering how **complexity** can be provided in the domain of nutrition, designers might identify opportunities for feeding schedules in which the mechanism of exercising control over feeding varies, as well as the type of food, thereby gradually increasing the level of variety and challenge that the animal experiences over time. Feeding in social settings also opens opportunities for experiencing and navigating complexity as the animals would in the wild. Increasing complexity can be an important dimension of incrementally augmenting animals' competence, and so careful consideration should be given to the rate at which greater complexity is introduced to provide optimal levels of safety and challenge.
- Taking a broader view, consideration would also be given to other intersections between **competence** and nutrition, which might lead designers to identify opportunities to promote behaviors, skills, physical strength, dexterity or speed that the animal's wild counterparts must possess to obtain food in native habitats.

**Table 1:** The welfare through competence design opportunities matrix.

		**Mental wellbeing**			
		**Nutrition**	**Environment**	**Physical** **health**	**Behavioral** **interactions**
**Competence**					
	**Choice**				
	**Control**				
	**Variety**				
	**Complexity**				

### Contextualizing and Prioritizing Design Opportunities

The WtC Animal Objectives Canvas (Figure 2) provides a tool for gathering the information required to effectively fill out the WtC Design Opportunities Matrix, and for prioritizing the opportunities to define objectives for a design project. In considering each component of the matrix, designers should take account of **the animal species**, the **context**, the **welfare goals of the population** and the **welfare goals of the individual animal**. In this context, “goals” refers to the needs and wants of the animal and are associated with improved welfare outcomes. Through this process some components will be responsive to high priority welfare needs of the animal. Conversely, some components may not be relevant to the needs of the animal.

#### Animal Species

Species-specific needs, goals and capacities should play a large role in identifying and selecting design opportunities. Designers should take account of the motivated behaviors, cognitive capacities, and environmental preferences of the species, as well as their abilities and preferences in terms of physiology, sensing, locomotion, and interaction with objects. For many species, there are known welfare challenges with managed care. For example, spatial considerations to enable captive snakes to adopt straight-line body postures (100); elevated positioning of features for arboreal animals (101); optimal enrichment provision for different life stages (suckling piglets, weaning piglets, and fattening pigs) of group-housed pigs on farms (37); and supporting Asian elephant herd dynamics in zoos with larger and more complex habitats (102). Designers should take account of these species-specific characteristics through reference to relevant literature and species experts with emphasis on characteristics observed in the wild.

#### Context

Design opportunities should take account of specific environmental factors which impinge on the animal's wellbeing. These may include contextual opportunities and constraints such as diurnal or nocturnal illumination, microclimate, space, physical structures and substrates, which may influence the extent to which an animal can express highly motivated behaviors such as exploring, play, and foraging behaviors such as rooting and digging, or hunting. The extent of environmental complexity and variety have been demonstrated to influence the degree of cognitive stimulation and challenge that can be provided to the animal (96, 103, 104) [See Coe and Hoy (70) for examples of contextual, built-in environmental enrichment]. Per Mellor et al. (7), the visual, auditory, and olfactory environment may have either a positive or a negative impact on wellbeing; for example, for some species, the sounds and presence (including position overhead) of humans or other species may elicit fear (105, 106). However, environments which offer visual and auditory variety, especially access to distant views, sounds and smells, may be enriching. Opportunities for design can be identified and prioritized by considering the ways in which the animal's environment 1) may negatively impact on wellbeing, 2) constrains existing welfare provisions, 3) already meets welfare needs.

#### Population Goals

Characteristics of the group that an animal is part of will shape welfare goals at several levels. Animals housed in groups are likely to have common welfare needs, goals and constraints. Accordingly, animal welfare is often considered at the group or population level (107, 108). The social characteristics of the group may also impinge on the welfare status of the group as a whole: factors such as the number of co-housed individuals, social hierarchies, ratio of male vs. females, and age composition of the group may impact welfare.

#### Individual Goals

The welfare needs of an animal, and ways in which those requirements can best be addressed, are also shaped by individual factors such as the animal's age, sex, personality, social relationships, and prior experiences. Older animals may have fewer opportunities for positive experiences due to declining physical or cognitive abilities, associated behavioral change or physical pain (109). Aging and life-stage have been found to significantly impact on the extent to which animals make use of enrichment (46, 110–112). As animals age, they may require tailored husbandry, enrichment and training, informed by ongoing monitoring of physiological, behavioral and cognitive changes (109). Sex has been associated with differences in welfare factors such as fearfulness (113) and participation in enrichment sessions (110). Personality traits such as aggressiveness, fearfulness, and risk-taking can impact on an animal's welfare and this may also be relevant in the case of traits such as sociability and nurturing behaviors (107). Many opportunities for improving welfare through animal-computer interaction may stem from the ability to use computational approaches to gather information about individual animals and automatically personalize interventions to individual animals.

### Defining Animal-Centric Design Objectives

The design opportunities identified and prioritized using the WtC Design Opportunities Matrix can be distilled to define design objectives: clear, measurable, specific statements of what a design intervention is intended to achieve in terms of animal wellbeing. The animal-centric design objectives, balanced with human-centric objectives, will provide input to the first cycle of an interaction design project. In many projects, selecting a single, high-priority design opportunity from the matrix will provide greater chances of overall success. As an example, identifying opportunities to increase environmental variety for zoo-housed primates might give rise to the objective to *create an interactive enrichment installation allowing animals to access a range of visual or audio stimuli*, as in the case of the Kinecting with Orangutans project (114) and the SakiTunnel installation (69). In some cases, secondary objectives may be included through this filtering process. For instance, a project to create interactive installation for primates might include secondary goals related to increasing primates' environmental control or promoting greater complexity of behavior (115). However, design teams should be aware that including too many disparate design objectives can lead to a lack of focus, and risks overloading a project with unrealistic aims.

A core challenge in projects of this nature is to explore how animal-centric and human-centric design objectives can be achieved simultaneously, through careful design. The interests and constraints of human stakeholder involved in the care of animals will need to be included as human-centric design objectives. Many design tools and processes exist for establishing human-centered design objectives and defining project objectives as they relate to an organisation's strategic goals. This framework therefore does not address that aspect of the design process. For example, in designing for zoo-housed primates, ACI researchers have identified the need to consider the varied requirements of zoo personnel and visitors (116), such as eliciting visitors' empathy for animals, while also providing meaningful education and enjoyment (114).

Animal-centric and human-centric design opportunities should also be used to define evaluation criteria. Through this process, designers are likely to identify several criteria for success, which may reflect the overarching goal of creating positive outcomes for animal and human stakeholders or, at a minimum, seek to ensure that the intervention has no negative impacts. For example, an initial summative evaluation of Kinecting with Orangutans measured its behavioral impacts, finding that the installation had no negative welfare impacts (46), while impacts on visitor perceptions of the animals were examined separately (114).

### Applying Interaction Design Process

Below, we describe a step-by-step process for using the WtC framework as part of an interaction design project. The process is offered as a way for teams of designers and animal carers to collaborate in creating interactive technology which prioritizes animal welfare objectives. This process responds to the real-world issues and conceptual challenges of designing for animals that we have encountered in our own work, and which are reported in the literature. We suggest that teams should conduct multiple iterations of this process cycle to gain greater clarity about animal needs, and include animal and human stakeholders as design participants.

#### Step 1: Understand Animals' Needs and Wants

1. Collate information to understand the needs and preferences of the animal, the context and current welfare provisions. The WtC Animal Objectives Canvas (Figure 2) provides a guide to collating such information to understand animals' needs and how they might be addressed. This approach can be used to address welfare needs in one of the Five Domains, or to address a known issue, or within a project that has broader goals, and which might impact on animal wellbeing. A broad, rapid survey of the types of knowledge held by different fields and practitioners can be valuable as a first step and allows for subsequent “deep dive” into the topics most relevant to design directions pursued as the project progresses.

1.1**Animal Species**. Gather information on the needs of the animal species and known welfare challenges in managed settings. This can be in the form of scientific and reliable popular publications; accounts, video recordings and data on wild populations and their habitat use; information from independent species experts such as veterinarians, animal welfare scientists and animal carers. Designers might employ a modified Delphi process, as suggested by Veasey (63, 64) to gather expert opinions on the relative importance of the behavioral and psychological needs of wild species in captivity. It is essential for designers to gather rich information from varied sources about the species' needs, behaviors and relative importance of welfare goals to avoid the pitfalls of creating products which have little value to the intended users (57, 117).
1.2**Context**. Gather information about the existing or proposed environment and its known impact on animal welfare. This might be collected through site audits of animal welfare (for example using the Five Domains methods), or through observations or reports on the animal environment. Designers can build on existing knowledge about how to address animal welfare challenges in sites such as zoos (35) working dog kennels (118), farms (119), and slaughterhouses (120). When designing for zoos, note that context includes both on-display areas and off-display areas (121). To understand opportunities and constraints on changes to the animals, collect information about the physical space in which the animal is housed, in terms of dimensions, physical structures and substrates, air flow, and temperatures. Additionally, information about the visual and auditory environment and other sensory aspects (e.g., odors from cleaning chemicals) might also be relevant to the animal. Understanding routines, schedules and protocols for feeding, health checks, cleaning, enrichment, training and other forms of interaction with familiar humans is important. Further, collecting details about any other activities which entail changes to the animal's environment, such as relocations, introduction of other animals, or changes to the presence of humans. For these events and routines, record occurrence (for example, any events which trigger them), frequency and duration or, ideally, obtain baseline data covering such factors.
1.3**Population Goals**. Gather information about the welfare needs of the group or population in which the animal is located. This might take the form of formal animal welfare audits and plans, or informal assessments from carers or handlers. Understanding the history for a specific population, for example through interviews with carers, can offer insights into important social dynamic nuances and previous enrichment successes or failures. This in turn, may influence identification of preferred opportunities and strategies. Peer reviewed literature can also provide insights into the welfare needs of other groups of the same species housed in similar settings. To identify related design opportunities, collect information on the social interactions between conspecifics, including the nature of such interactions, and whether they entail positive or negative experiences for the animals involved.
1.4**Individual Goals**. Gather information about the welfare needs of the individual animal. This might include the outcomes of existing animal welfare audits and reports. Where possible, up-to-date “baseline” assessments of the animal should be conducted, which will be valuable when evaluating success. Another option is to include light-weight assessment techniques, such as the qualitative “free choice profiling” approach proposed by Wemelsfelder et al. (122). To understand the design opportunities and constraints pertaining to an individual animal, gather behavioral data such as the animal's current activity levels, use of enrichment, interactions with conspecifics, human carers and others, and feeding preferences. This may be obtained through direct observation, interviews with carers and video recordings—including hours when carers are not present—which can be analyzed by carers, species specialists or welfare specialists. Comparing an individual's behavioral data with that of conspecifics from the same or different site may be revealing. Consider the likely physiological, behavioral and cognitive impacts of maturation or aging, and other future changes such as pregnancy and rearing young.

#### Step 2: Identify Animal Centric Design Opportunities

2. Consider in turn each of the focus areas defined by the Coe Individual Competence Model (Choice, Control, Variety, and Complexity), to identify ways in which the animal's situation might be changed for the better, if a design solution could be successfully realized.

2.1 Consider how enhancing **Choice** might improve the animal's situation within the welfare domain currently under scrutiny. This might include interventions which allow the animal to choose between a wider range or frequency of options, or throughout a 24-h cycle and not just when care staff are present (123, 124). Consider how increased choices could enhance the animals' ability to communicate preferences, or expand the areas of life in which the animal can exercise control, engage complexity and build competence (14, 125).
2.2 Consider how greater **Control** for the animal might allow for better welfare outcomes in this domain. An important consideration is whether an animal can be given continuous control over basic aspects of their situation, including freedom of movement between cooler/warmer, dryer/damper, lighter/darker, open/enclosed/novel/familiar spaces (76, 126). Interventions which allow appropriate levels of control over when and how to access essential provisions such as water, food, shelter, conspecifics, and enrichment might play an important role in addressing welfare needs (70, 127). Systems which allow animals to control their environment (e.g., lighting, temperature, water or sprinklers, sound, breeze) and activities to train animals to use such systems would also be examples of designing for greater animal control (70).
2.3 Consider how increased **Variety** of opportunities can be offered, Variety may be considered in types of food, varieties of environments, methods of physical conditioning opportunities, or companions (74). Variety also may be considered in other dimensions or combination of dimensions such as times of day, frequency, duration, and location of activities or rest areas or changing seasonal conditions for example.
2.4 Identify ways in which **Complexity** can be introduced through combinations and permutations of existing, or new, interventions. This might include complexity of environments and multisensory stimuli (128). Social interactions with larger animal groups and housing with other species are inherently complex. Complexity of nutrition can include variation by time and by seasonally changing natural conditions, which has been shown to be beneficial to animal health. Complexity should be considered carefully when introducing mental challenges, such as cognitive enrichment: designers should ensure that appropriate levels of challenge are provided to individual animals, and that complexity is incremented at an appropriate rate to build competence without causing detrimental levels of frustration (129).
2.5 Consider, more broadly, how an animal's **Competence** can be augmented through design and deployment of interventions. In this, consider timing and building on the individual animal's prior experience. It may be valuable to consider at this stage how the prototyping process will be used to introduce animals to novel apparatus, new forms of interaction, and new behavioral opportunities.

#### Step 3: Define Animal-Centric Design Opportunities

3. Prioritize design opportunities and distill them to define animal-centric design opportunities.

3.1 Prioritize the opportunities identified, according to likely positive impacts on the animal's overall wellbeing.
3.2 Select a small number of opportunities, ideally 1 or 2, to be addressed in the design project.
3.3 State design objectives unambiguously, in a framing which makes it clear which animal needs are reflected, and the overarching rationale for the design objective.
3.4 Identify evaluation criteria associated with the selected design opportunities. Determine how evaluation will be conducted, by whom, and how the design team will know if the aims have been met.

#### Step 4: Define Project Design Objectives

4. Identify initial design objectives for the project, and evaluation criteria.

4.1 Identify human-centric design objectives. We anticipate that organizations may have access to existing processes for conducting human-centered design and eliciting organizational requirements for design projects. We note that it is important to consider the goals and constraints impinging on caregivers, organizational stakeholders and perhaps other groups (e.g., the broader public). It is valuable to acknowledge how existing systems, organizational or societal values and commercial considerations constrain the range of options, and discuss how the design thinking might be expanded if these limitations did not exist. There is also a need to consider how design might impact on human attitudes to animals. For example, in zoos, interventions can be designed to support educational aims and promote positive attitudes to animals (114, 130). But technologies can also inadvertently foster misunderstandings about animals' needs or negatively impact caregiving (58, 131).
4.2 Identify potential conflicts between the goals of human and animal stakeholders and determine how these tensions will be managed. In some settings, such as agriculture, the conflicting pressures of organizational drivers and competing perspectives of different stakeholder groups can mean that attempting to make changes to improve animal welfare presents a “wicked problem” (132). Iterative design thinking can provide a valuable approach to addressing wicked problems (133), allowing for reframing problems and identifying novel solutions as well as new ways of working which respond to conflicting goals (134).
4.3 We suggest that it is valuable to be explicit about which stakeholder will benefit from a stated project requirement. For example, user stories provide a useful framing for articulating design objectives from the perspective of a specific stakeholder, making clear how the goal is relevant to their overall wellbeing or objectives. User stories follow a prescribed format: “As a [stakeholder type] I [want to], so that [….]”.

Example: “As manager of a primate colony in a biomedical research facility I want to find entertaining and diverting activities for macaque monkeys so that they are quiet and calmly occupied while they are isolated and closely confined during lengthy biomedical testing procedures”.

#### Step 5: Ideate to Identify Alternative Solutions

5. Gather ideas and inspiration for alternative design approaches from multiple sources and conduct a range of ideation activities to explore the problem from new perspectives. When conducting ideation, project teams should make use of the information that has been gathered about the animal's needs and wants at Step 1. Alternative solutions are likely to start by responding to the design opportunities identified at Step 2, but may extend more broadly as new ideas are gathered, and as new insights about the problem space are generated.

5.1 Practitioners who work primarily in animal sectors can benefit from learning about the capabilities of emerging technologies by reviewing white papers and technology sector magazines, or through connecting with university researchers. Inspiration may be drawn from technological interventions in other domains: for example, interfaces for primates have been inspired by installations in public spaces and for young children. Learning some of the ways wild animals use their habitats is often inspiring.
5.2 Ideation activities can include brainstorming in groups or individually, sketching, storyboarding, soliciting ideas from a wide audience, mind-mapping and deliberately exploring problematic ideas (“worst possible idea” brainstorming). A deck of cards, such as the “Concept Craft Cards” created by French et al. (62) can support groups to generate ideas by prompting them to consider different aspects of a problem and alternative approaches. Several guides and templates for conducting ideation activities and “design thinking” are available online, from organizations such as the Stanford d.School, Google and IDEO. Including a wide range of stakeholders in ideation activities can help to broaden the thinking and examine the problem from alternative perspectives.

#### Step 6: Develop Prototypes

6. Create and deploy prototypes to investigate the likely effectiveness of a proposed intervention and barriers to its implementation.

6.1 At early iterations of the design process, create “low fidelity” prototypes such as non-working hardware prototypes and partial prototypes to gain feedback from animal users and stakeholders. Early prototypes can be rapidly created with the aim of determining whether the proposed design objective will contribute to animal wellbeing as envisaged. One approach is to use the “Wizard-of-Oz” technique (96, 135), in which a human operator provides the interactivity or effects which will be delivered by a computerized system. This approach will help designers to avoid spending excessive time and resources on developing a solution which is not attractive to animals or does not meet their interests. In addition, early prototypes can be designed to minimize animal training needs and put aside non-functional requirements (such as robustness and longevity) in order to quickly and cheaply determine whether the animal will benefit from the changes or behavioral opportunities that the intervention delivers.
6.2 A key tenet of the interaction design process is that prototypes should be created to be thrown away. When designing with animals, this means that prototypes should also be designed so they can be safely destroyed by the animal users. Designers should therefore repurpose materials and objects which are known to be safe for the target animals, and avoid deploying computing components which could be chewed or ingested. Using familiar objects and materials is also likely to reduce the impact of the “novelty effect” and neophobic responses on animals' initial interactions. When prototyping for animals, “decomposition” (96) provides an approach to examine different aspects of design separately. For example, physical hardware components of a proposed device can be constructed and given to animal users to see if they are usable, and ascertain whether animals will need to be trained in how to operate them.
6.3 At later iterations of the design process, deploy “higher fidelity” functional prototypes which progressively approximate more closely the fully working ACI intervention. Designers should defer investing in high-cost, high-complexity prototypes until sufficient evidence has been gathered that the design objective will have a positive impact on animal wellbeing, and that the proposed design will be effective in achieving that goal. This approach allows design teams to be responsive to data gathered during the design process, including making fundamental changes to the design approach, and shifting the design objectives if required.

#### Step 7: Evaluate Against Design Objectives

7. At each iteration of design, prototypes should be evaluated against the design objectives established in Step 4. Evaluation can reveal new insights about the needs and preferences of animals and humans. This knowledge, if captured, can be valuable in its own right, and also inform future design projects.

7.1 “Formative evaluation,” conducted throughout the project, can allow designers and stakeholders to improve on the design. In early iterations of the process, Wizard-of-Oz and prototype decomposition techniques can allow for evaluation which focuses on assessing (a) to what extent the design objective provides a valid pathway to enhancing animal welfare and (b) to what extent the design is likely to be successful in meeting the design objectives. Qualitative evaluation with stakeholders, such as video review and focus groups, will allow designers to learn more about the needs of animals and humans, and about potential barriers to successful deployment. This approach allows for refining or changing the design objectives 1) if initial goals impractical or unachievable, 2) if better opportunities are discovered, or 3) if animals' responses to prototypes reveal new directions for design.
7.2 In later stages of the project, once design objectives and the overall design approach have been validated, evaluation can be expanded to assess and improve on other aspects of design such as usability (for animals), functionality, performance, ease of deployment (for human carers), robustness, reliability, and maintenance requirements.
7.3 At a final iteration of the design cycle, a complete working prototype should be used to conduct a summative evaluation, to collect data about the extent to which the intervention is successful in achieving the design objectives. This data will provide a baseline for ongoing evaluation of the long-term effectiveness, and provide a valuable resource for other organizations seeking to deploy a similar system. Evaluation which seeks to make claims about the effectiveness or welfare impacts from the animal's perspective should use appropriate methods, informed by animal behavior and welfare science (56, 98). It is likely that a reliable, robust prototype will be required for this evaluation, and that design changes should not be made once the study has commenced.

## Anticipated Results

The WtC framework can be used by diverse sectors to guide design with and for animals and capture learnings. Here, we present three scenarios to illustrate how the framework might be used to support design projects in zoos, animal production, and companion animal care.

### Application in Zoos and Sanctuaries

The WtC framework can be used by zoos and sanctuaries to enhance and capitalize on their existing expertise in designing and creating animal enrichment, and to support effective reuse in other settings.

*Scenario: Designing to elicit birds' natural behaviors in acquiring food*.

*A zoo holding passerine (perching) birds seeks to encourage the birds' natural behaviors and problem-solving abilities for locating and extracting food. The birds' natural habitats are relatively complex and varied, presenting diverse challenges in locating and extracting food, but existing zoo enclosures lack such opportunities*.

The WtC Animal Objectives Canvas prompts designers to weigh the needs and attributes of the species, the individual animal and population, as well as the zoo context and the behavioral opportunities it provides. In the wild, most passerine birds live in relatively complex naturalistic environments which present diverse challenges for obtaining food. Many bird species, including those of the corvid (crow) and psittacine (parrot) families are naturally intelligent and curious. Table 2 shows an example of how the WtC Design Opportunities Matrix might be used in addressing this scenario, identifying how greater choice, control and variety and complexity can all contribute to offering richer behavioral opportunities related to “working” for food. The matrix also reveals how this goal intersects with environmental, health, and behavioral domains of welfare. To meet the behavioral needs of intelligent birds housed in an environment which lacks complex foraging opportunities, designers might decide to prioritize design opportunities related to increasing the complexity of food acquisition tasks, as highlighted in Table 2. By working through the WtC Animal Objectives Canvas, designers will have acquired a deeper understanding of relevant characteristics of the animals and their environment, and will have identified additional needs which might be incorporated as secondary design objectives. The WtC Interaction Design Process will guide designers to consider how the objectives might be met using alternative technologies, such as computerized puzzle feeders, automated scatter feed devices.

**Table 2:** WtC design opportunities matrix used to identify opportunities for increasing natural feeding behaviors of passerine birds housed in a zoo.

		**Mental wellbeing**			
		**Nutrition**	**Environment**	**Health**	**Behavior**
**Choice**	Opportunity to choose between alternative foods	More and different features related to foraging to choose from	Reduce self-harming behaviors (e.g., overgrooming) through increasing foraging	Choice in food acquisition behavior
**Competence**	**Control**	Agency in feeding, including timing			Agency in acquiring food, including timing
	**Variety**	Wider variety of foods	Greater variety in environmental features related to foraging		Greater variety of food acquisition tasks
	**Complexity**	Complexity of diet, including weekly or seasonal variance	Greater complexity of environmental features related to foraging	Muscular fitness for food acquisition	Greater complexity of food acquisition tasks

*Bold border indicates opportunities identified as high priority for animal welfare*.

### Application for Animal Production

For livestock production, the WtC framework can be used to incorporate animal welfare objectives into the design of computerized systems, now being widely deployed as part of precision livestock farming initiatives. This will support the sector in paying increasing attention to positive animal wellbeing as a contributor to productivity, and to public concerns about farm animal welfare. In addition, ACI interventions can be used to capture data about animals' interactions and movements, supporting the inclusion of animal welfare and behavior metrics in precision farming systems.

*Scenario: Designing to enable cows' self-grooming behaviors*.

*Self-grooming is an important natural behavior in farm animals such as cows, which is vital for health and which appears to be enjoyable and highly motivated, especially when animals are restrained* (136). *In environments such as freestall barns, cows make extensive use of fences, walls and pen objects for scratching and grooming. However, such objects are not sufficient for all self-grooming behaviors that cows want to perform* (137).

Using the WtC Animal Objectives Canvas would prompt designers to consider the ways in which cows' self-grooming is important for physical health, is part of social behaviors, and may be a self-soothing behavior for coping with stress. Freestall barns generally offer little structural variety, and are likely to offer cows few objects for scratching against. Additionally, the size, texture and shape of such objects might not be sufficient to allow for satisfying scratching of different body parts. Table 3 shows an example of how designers might use the WtC Design Opportunities Matrix to address this scenario. In this example, opportunities have been prioritized (as highlighted in Table 3) for offering greater control and variety for self-grooming to contribute to physical health and mental wellbeing, which will inform the animal-centric design objectives. Through the WtC Interaction Design process, the design team might examine how these objectives can be reconciled with the need for efficient, easy to clean facilities. Ideation might lead designers to explore how mechanical brushes can be improved on to provide access to a wider variety of textures and surfaces, or provide a wide variety of pressure and speed of brushing by responding to pressure and movement.

**Table 3:** WtC design opportunities matrix used to identify opportunities for enabling cows' self-grooming behaviors.

		**Mental wellbeing**			
		**Nutrition**	**Environment**	**Health**	**Behavior**
	**Choice**		Choice of objects and surfaces to interact with in the environment	A choice of ways to meet grooming and scratching needs	Ability to choose between grooming-related behaviors
**Competence**	**Control**		Agency over when and how to interact with different objects and surface	Ability to groom and scratch at will, e.g., in response to itches or for stress reduction	Freedom to perform grooming and scratching behaviors at any time
	**Variety**		Wider variety of grooming related objects and surfaces	Ability to groom all parts of the body	Allow for greater variety of grooming/ scratching behavior. Allow for individual preferences
	**Complexity**				

*Bold border indicates opportunities identified as high priority for animal welfare*.

### Application in Companion Animal Care

With growing interest in digital technologies for pet care and enrichment (such as video call systems and robotic toys), the WtC framework can guide the design and use of devices to deliver wellbeing benefits to domestic animals based on specific needs and objectives. Applying the WtC framework can help allay concerns that some pet care devices are designed primarily to appeal to the concerns and motivations of owners, rather than addressing genuine wellbeing issues affecting companion animals.

*Scenario: Improve pet dogs' experience of their sound environment*.

*For some pet dogs left alone during the day, external sounds can be a source of stress or distress. For others, sound can be an important form of varied environmental stimulation, and individuals have distinct preferences in music genre, for example* (138). *While free-ranging dogs can select or modify their own sound environment, for example by moving to a different resting place, dog companions confined in homes or yards are unable to do so*.

Using the WtC Animal Objectives Canvas to investigate this issue reveals several ways in which pet dogs can benefit from wider variety of audio stimuli. For dogs housed in urban or loud environments, external and unpredictable noises may be stressful, so a sound environment which masks such noises may be beneficial. For other dogs, sound may be a form of enrichment in another wise monotonous setting. Seeking out alternative sound environments may constitute a valuable form of environmental exploration for dogs who are confined. Table 4 shows an example of how the Design Opportunity Matrix might be used to address this scenario, and indicates that in this instance designers have given greatest priority to animals' control over their auditory environment. As part of a design project with the objective of allowing dogs to change their auditory environment, designers might explore the possibility of using different types of sensors (e.g., proximity sensors, activity monitors) to provide dogs with alternative sound environments which the dog can choose between by moving from one area to another, and which vary according to the dog's level of movement and wakefulness.

**Table 4:** WtC design opportunities matrix used to identify opportunities for improving pet dogs' sound environment.

		**Mental wellbeing**			
		**Nutrition**	**Environment**	**Health**	**Behavior**
	**Choice**		Choose between alternative auditory environments or stimuli		Ability to choose environments suited to e.g., resting, sleeping, interactivity
**Competence**					
	**Control**		Ability to change the auditory environment	Ability to minimize exposure to distressing auditory stimuli	Ability to rest, sleep, interact etc. when desired
	**Variety**		Greater variety of auditory stimuli, less predictability		
	**Complexity**		Greater complexity of auditory environment to avoid habituation		

*Bold border indicates opportunities identified as high priority for animal welfare*.

## Discussion

The WtC framework, by providing a practical approach to centering animals in technology design projects, will prove useful to ACI researchers and practitioners who seek to improve animals' lives. In our presentation of the framework, we provide definitions, processes and examples which will support collaboration and the exchange of ideas and approaches between the various disciplines, expert and stakeholder types involved in successful animal-computer interaction design projects. This paper also delivers a robust response to long-running debates in the field of ACI about the feasibility of including animals as stakeholders, by outlining a design process which can capture and respond to the interests of animals, in turn offering them a good life.

### Enabling Animal-Centric Design Practice

While interaction design and “design thinking” approaches are now widely used for human-centered innovation, the WtC framework provides a much-needed structured approach for project teams to include current understandings of animals and their needs. The framework enables interdisciplinary collaboration on this topic by providing a conceptual frame for understanding the different types of knowledge that can be brought to bear in an ACI project, for sharing those different forms of knowledge across the team, and for understanding how they intersect with each other and support the project. In addition, the WtC Animal Objectives Canvas provides a tool to guide teams in identifying what it is they need to know about the animal and its world, while the design process indicates how that knowledge can be applied and further developed through the project.

There is considerable divergence between the aims and practices of different animal sectors, and between the needs and objectives of the human stakeholders that interact with them. Furthermore, we anticipate that many organizations will have established processes for eliciting, documenting and validating project requirements, which will provide input in the form of human-centric design opportunities and objectives of the WtC interaction design process. While the WtC framework can capture and respond to diverse sectorial needs, we recognize that for some organizations, it will be most beneficial to use the WtC Animal Objectives Canvas as inputs to IT project management processes.

### Advancing ACI Debates and Scholarship

A core strand of ACI scholarship engages with the question of “to what extent design processes can reflect the needs of animals as stakeholders and users?” (59), and how human-centered interaction design methods can be adapted to achieve this (1, 3, 55). Attempting to place animal stakeholders at the heart of design work raises a range of methodological challenges (55), including the issue of identifying appropriate design objectives, aligned with the animals' interests and welfare needs (2, 59). In this paper, we have proposed a framework which offers a new pathway to progress this dimension of ACI theory, building on animal welfare science theory and design approaches developed in zoos and sanctuaries. This provides a structure that addresses methodological issues of ACI design and provides a foundation that can support designers to avoid the pitfalls of anthropocentrism (59) and inadvertent negative welfare impacts (57).

The framework expands ACI's interaction design methods by building on well-established concepts of animal welfare science and design techniques developed over several decades in zoos and sanctuaries. As we have illustrated, the WtC framework provides a model which can be applied in any animal management setting. The model we offer foregrounds animal-centric objectives, acknowledges that human stakeholders may have competing objectives, and indicates how both sets of objectives can be incorporated into an interaction design project. In this way, the WtC framework constitutes an important advance for the field of ACI by providing a methodological basis for design projects that are well-informed about target animals, their species and context, to be able to contribute to a good life for animals.

### Enabling Interdisciplinary ACI Research and Education

Creating technological interventions for animal wellbeing is inherently interdisciplinary work, which can entail collaboration between designers, computer scientists, species specialists, animal welfare scientists, carers familiar with the group and individual animal, and other stakeholders with knowledge of the context and organizational aims. Several ACI scholars have drawn attention to the need for ACI scholars to work closely with specialists in animal behavior and welfare (56, 57, 98), and to understand how to elicit and apply different types of expert knowledge about animals (96). The WtC framework provides a structured approach to achieving this and offers practical guidance to researchers and technologists who are new to animal-centric design. Conversely, the visual components of the framework (Figures 1, 2) also supports ACI researchers to communicate with scholars from other disciplines about the interaction design process. This illustrates how animal centric knowledge can be incorporated into interaction design, and how design work can lead to deeper understandings of animals' needs and wants, as well as the production of a technological artifact. Our hope is that this approach will enable non-ACI specialists to envision ways in which technology and animal-centric design can further their efforts to increase the welfare of animals in their care.

The WtC framework supports ACI scholars to resolve tensions between iterative interaction design processes with the empirical methods of animal-related sciences. While rapid prototyping favors innovation and responsive design, these agile approaches are not compatible with the qualitative, ethological evaluation methods generally used by animal behavior and welfare researchers to assess the effectiveness of an intervention (96). To address this, the WtC design process proposes a distinction between lightweight “formative” prototyping, conducted to inform subsequent cycles of design, and rigorous “summative” prototyping, which might deploy ethological methods to assess the effectiveness of a finished artifact. An important facet of the framework is the focus on defining and refining animal-centric design objectives, to guide both formative and summative evaluation and ensure the project retains sight of the animal welfare goals.

With computer science and design students showing increased interest in ACI, important challenges for educators are to sensitize students to animal welfare in iterative development, and to offer guidance in methods for eliciting animal-centric requirements (55). The WtC framework provides a structured foundation and step-by-step process which responds to this need, offering a tool for training ACI students in applying interaction design principles to animal-centric work, and a foundation for cross-disciplinary projects with animal scientists in any managed animal setting.

### Future Research to Expand the WtC Framework

Envisaged benefits and contributions of the WtC, as discussed above, will be expanded with further data about the design journey of projects undertaken using these tools. The WtC tools and process provide structured prompts for design teams to capture project objectives, decisions, and readjustments, as well as the results of prototype evaluations. Technology interventions often entail unexpected outcomes and unanticipated consequences (139), and documenting projects' design journeys and lessons learned will provide valuable insights for future design projects, for ACI research, and for enhancing the WtC Framework and associated tools.

An important aim of the WtC framework is to bring to the fore the tensions between human-centric objectives (including organizational aims and commercial considerations) and the animal-centric design objectives that emerge. However, techniques for addressing issues of ethics and power in ACI (59) are left to the discretion of designers. As the WtC framework is adopted by different animal sectors, it will be valuable to investigate how specific tools and approaches can aid designers in identifying solutions to provide animals with a good life which simultaneously address the needs of human stakeholders and organizations.

## Conclusion

The WtC framework integrates existing, best-practice models and process, to create a structured guide for designers to create interventions that respond to animals' needs and wants, and mitigate the risk that human-centric aims prevail over the interests of animals. In the WtC Animal Objectives Canvas, designers are provided with a novel tool which leverages contemporary theory and best practice to aid them in understanding what animals need to live a good life, and for identifying relevant design opportunities and objectives. We provide a structured approach for iterative interaction design that can lead to deeper understandings of what animals need and want, allowing for refinement of animal-centric design objectives as well as creation of a technology product. The WtC framework is presented to provide a practical tool that can support collaboration and communication in interdisciplinary teams, providing a foundation for better design. Acknowledging the diverse needs and practices of different sectors that involve animal management, the WtC framework is widely applicable and flexible to satisfy the needs of different animals, organizations, and settings, and can be complemented with other organizational toolsets and protocols.

By presenting a framework that integrates models of animal welfare, design for animal competence, and interaction design process, this paper responds to core challenges and debates related to animal-centric technology design. Crucially, the WtC framework contributes new thinking and conceptual approaches to the core ACI challenge of centering the animal in design, supporting a good life for animals.

## Data Availability Statement

The original contributions presented in the study are included in the article/supplementary material, further inquiries can be directed to the corresponding author.

## Author Contributions

The conceptualization of this work and development of the WtC framework we present was shared predominantly between SW (45%) and MC (35%). JC contributed to the framework development (20%) by providing feedback and expertise on the Coe individual competence model. Writing and preparation of the manuscript was conducted predominantly by SW (60%). MC wrote sections of the manuscript (30%) pertaining to animal welfare science. JC provided writing contributions (10%) to sections of the manuscript on animal enrichment and zoo-based design. All authors contributed to the article and approved the submitted version.

## Conflict of Interest

The authors declare that the research was conducted in the absence of any commercial or financial relationships that could be construed as a potential conflict of interest.

## Publisher's Note

All claims expressed in this article are solely those of the authors and do not necessarily represent those of their affiliated organizations, or those of the publisher, the editors and the reviewers. Any product that may be evaluated in this article, or claim that may be made by its manufacturer, is not guaranteed or endorsed by the publisher.
